# Supplementary material for: Trans-driven variation in expression is common among detoxification genes in the extreme generalist herbivore Tetranychus urticae
Source: PLoS Genet. 2022 Nov 14;18(11):e1010333. doi: 10.1371/journal.pgen.1010333 (PMC9704763; doi:10.1371/journal.pgen.1010333)
Supplement: Attachment — Submitted filename: Kurlovs_2022_comments.docx [file pgen.1010333.s015.docx]

**Overview**

The manuscript “Abundant trans-driven variation in detoxification gene expression in the extreme generalist herbivore Tetranychus urticae” by Kurlovs et. al considers the mechanism of gene regulation in spider mites with a focus on genes involved in host adaptation and acaricide resistance. By measuring allele specific expression in F1 crosses, they identify trans regulatory factors as playing a predominant role in shaping the transcriptome, particularly in genes known to play critical detoxification roles.

The manuscript is well written and technically sound. It further makes fundamental insights to the nature of molecular adaptation; the reviewer plans to use this in their teaching because of its high quality. I therefore happily recommend its publication in PLOS genetics. The reviewer, however, does have several comments which should be addressed prior to publication in addition to more minor corrections and suggestions.

**Major comments:**

*Length and readability*

The manuscript is technically sound, but it was partially obfuscated by the level of detail and the length. While sometimes this is unavoidable at others there seems to be superfluous information that could be relegated to supplementary material or a separate review. To take one example, Lines 177-221 describe in detail the resistance and host status of the strains, but this could probably be condensed in order to focus the paper on the more interesting findings presented. In the same content, the number of references 113 seems more appropriate for a review rather than a primary research article (e.g. in Line 83 are all references 12-21 necessary?). The manuscript would be greatly improved by streamlining the results/discussion to emphasize the (truly amazing) points that are discovered in this paper. There are suggestions that the reviewer has made in the “Minor comments” section, but full discretion is given to the authors.

*Categorization of gene regulation*

Based on the Figure 3, and the methodology description in lines 816-840, there did no appear to be any FC cutoff for classifying genes as “cis”, “trans” etc. and this distinction is rather based purely on statistical differences in counts between the 5 biological replicates of alleles or parental lines. Are such small changes (<1Log2FC) biologically relevant? Has this approach (No FC cutoff) been used previously?

Further, the fact that placing such a FC threshold shifts in the proportion of “trans” regulated genes (Lines 314-317; Fig 4) including those present in the detoxification genes (Figure 3 E-H), it appears that mechanisms like “Cis” and “Synergism” play a substantial role in the regulation of gene with large changes. Again the question is of biological relevance, and the reviewer questions whether such an emphasis on “trans” regulation (e.g. the Title) is justified. While several interesting cases are cited of purely “trans” regulation at higher magnitudes (e.g. *Cyp392* family, DOGs), the focus on this within the manuscript seems to minimize the critical contribution of “cis” or “synergistic” mechanisms. It is quite possible the reviewer has missed a key argument, but the authors should address this in the text or explain in the rebuttal why this focus is justified.

On a related issue, it is not clear in in lines 816-840 how categories such as “trans”, “antagonistic”, and “compensatory” were distinguished from one another (only how “cis” and “trans” were distinguished). The authors should edit this to ensure clarify. From Figure 3, which only looks at FC it seems like there are many cases where categories appear to overlap although I assume this is because statistical significance only was used to make such distinctions

**Minor comments: (Reviewer text highlighted)**

*Lines 47-50:* “In particular, a clade of CYPs with documented broad substrate-specificity including multiple pesticides, as well as DOGs that have recently been shown to have broad substrate specificity against plant specialized compounds, were exceptionally highly upregulated as a result of trans effects (or in some cases synergism of cis and trans) in the most multi-pesticide resistant strains.”

*This sentence should be simplified to improve readability*

Lines 137-138: “and a high-quality genome, and is easy to inbreed”.

*Please improve the wording to avoid “and” twice*

Lines 162: “revealed 1.19 million high-quality variants”.

*It might be useful to include the density of SNPs in the genome. i.e. 90MB / 1.19 million variants = ~ 1 variant per 800,000 bp?*

Line 160-170: This can probably be moved to the methods

Lines 184-186: “MR-VPi and MAR-ABi, for which progenitor populations were previously reported to be multi-acaricide resistant, exhibited the highest levels of resistance to compounds in multiple acaricide classes (Fig 2A).”

*This sentence is repetitive and should be shortened*

Lines 177-194:

*This paragraph describing the resistance status of each line could probably be shortened or combined. Likewise the following paragraph (194-221).*

Lines 244-250 not super necessary

Lines 256-258: “and because test crosses revealed that viable F1 progeny were readily produced for each of the four crosses (genetic incompatibilities or partial incompatibilities are not uncommon among T. urticae strains [54,70–72]).”

*Were these genetic incompatibilities why the London strain was not chosen or was there another reason that a more standard line could not be used (e.g. mapping bias as it is the reference genome)?*

Line 278: “For consideration for inclusion in a given comparison”

*Delete “For inclusion”*

Line 310:

*The reviewer was unable to open Figure S2*

Figure 3:

*Perhaps I missed it, but it would be good to clearly state which direction + and – LogFC refers to throughout the ms. E.g. positive numbers always mean enrichment in the unique strains.*

Line 375 (Figure 5):

*It is unclear how the specific P450s were chosen to for analysis in the phylogeny as only 8 were present in the GO term.*

Line 400/Discussion: “effects, as observed for the DOG genes DOG1, DOG11, and DOG13 (in MAR-ABi) (S3 Fig), the lipocalins”

*Are these genes which show similar trans regulation close to each other physically on the chromosome. Could they possess common regulatory factors?*

Line 494-496: “with dominant versus additive inheritance closely mirrored the higher frequency of trans versus cis effects (this was true among comparisons as well; for instance, for the ROS-ITi x SOL-BEi contrast, relatively fewer trans effects and dominance were detected as compared to the other three, compare Fig 3A-D and Fig 4A to Fig 6A).”

*No need for parentheses, just make this an additional sentence.*

Line 555-574:

*Cap’n’collar is probably worth a mention in the context of potential trans acting factors as it has been widely cited as a transcriptional regulator in insecticide resistance.*

Lines 576-611:

*The authors could probably shorten discussion of specific P450s*

Lines 724-725: “we used the approach of Villacis-Perez et al. [54].”

*The authors should briefly explain the approach of Villacis-Perez.*
