# Supplementary material for: Trans-driven variation in expression is common among detoxification genes in the extreme generalist herbivore Tetranychus urticae
Source: PLoS Genet. 2022 Nov 14;18(11):e1010333. doi: 10.1371/journal.pgen.1010333 (PMC9704763; doi:10.1371/journal.pgen.1010333)
Supplement: Attachment — Submitted filename: 13092022plos_genetics_response_last_11AM.docx [file pgen.1010333.s016.docx]

Please note: Reviewer comments are in black, and our responses are in purple, with changed text in italics. We have uploaded a “clean” version of the manuscript, as well as one showing track changes for the benefit of the reviewers. No figures, either main or supplemental, are changed. Two new supplementary tables are added, and another modified, as described below.

Reviewer #1: The research paper by Kurlovs et al, entitled: “Abundant trans-driven variation in detoxification gene expression in the extreme generalist herbivore Tetranychus urticae” is a very interesting and comprehensive study on the regulatory mechanisms of gene expression in the major agricultural pest T. urticae. It provides important insights on the genetic basis of in xenobiotic resistance and plant adaptation mechanisms in T. urticae, and determines gene expression regulatory mechanisms that could be adaptable to additional agricultural pests. The methodological approach is sound, conclusions are supported by the study’s findings and the manuscript is very well written. I really have only minor comments to make that the author could consider for their revises manuscript: (1) the results of the study indeed highlight the importance of detoxification (and other) gene expression and their participation in the very high resistance levels encountered. in this respect it would be interesting to discuss the “translational” potential of these findings in operational terms; in other words, would it be possible to develop a molecular diagnostic that could be used for monitoring of these important mechanisms in field populations? (2) In line with my previous comment, how could these findings be “translated” to the development of new active compounds? (3) Since trans regulation seems to be the key mechanism for gene expression regulations it would be interesting to discuss in more details which these possible mechanisms could be (common transcription factors, epigenetic regulation- miRNAs etc).

We thank the reviewer for the appreciation and constructive comments. Our manuscript is obviously under review at PLOS Genetics, a journal that publishes many articles that are more “basic” than “applied” in their nature. However, point 1 is certainly well taken, and our finding of strong *trans* regulation for genes with broad substrate specificity (like CYP392 and DOG genes) does suggest that this molecular phenotype could be used in screening for strains that may have elevated resistance levels to many compounds. Hence, in the concluding paragraph of the Discussion now reads:

“*Among T. urticae strains we found that large changes in the expression of genes known or suspected to be important for detoxification or host plant use were often underpinned by variation in trans-regulatory environments. This was especially striking for genes encoding CYPs documented to detoxify multiple acaricides, and DOGs that can metabolize a range of plant-derived aromatic compounds. The extent to which high expression of genes encoding detoxification enzymes with broad substrate specificities can serve as biomarkers to assess the resistance potential of T. urticae strains warrants future investigation. More generally, our findings position T. urticae as an attractive system for future studies to characterize the molecular basis of the control of gene expression variation in xenobiotic and host plant use pathways.”*

With respect to point 2, our study further identifies genes differentially expressed between comparatively acaricide susceptible and resistant strains of *T. urticae*. As for any such study, genes with differential expression are potential targets for compound development.

With respect to point 3, the *cis*/*trans* experimental design we used cannot identify the exact nature of *trans* regulators, and certainly the range of possible regulators is large, as noted by Reviewer 1. We did discuss potential regulators for which there is some experimental support in *T. urticae* in the Discussion of the original submission, and this remains in the resubmission. Now, we also specifically mention Cap‘n’collar isoform C regulators and a review on transcriptional regulators is added to the Discussion, see also response to Reviewer 2; *Amezian et al. 2021*, PMID 33838715). However, we believe any more than this is overly speculative (and possibly would have to read more like a review). Such a discussion would also lengthen the manuscript, and two reviewers have explicitly asked that the manuscript be shortened (which we have done).

Reviewer #2:

**Overview**

The manuscript “Abundant trans-driven variation in detoxification gene expression in the extreme generalist herbivore Tetranychus urticae” by Kurlovs et. al considers the mechanism of gene regulation in spider mites with a focus on genes involved in host adaptation and acaricide resistance. By measuring allele specific expression in F1 crosses, they identify trans regulatory factors as playing a predominant role in shaping the transcriptome, particularly in genes known to play critical detoxification roles.

The manuscript is well written and technically sound. It further makes fundamental insights to the nature of molecular adaptation; the reviewer plans to use this in their teaching because of its high quality. I therefore happily recommend its publication in PLOS genetics. The reviewer, however, does have several comments which should be addressed prior to publication in addition to more minor corrections and suggestions.

We thank the reviewer for the appreciation and constructive comments.

**Major comments:**

*Length and readability*

The manuscript is technically sound, but it was partially obfuscated by the level of detail and the length. While sometimes this is unavoidable at others there seems to be superfluous information that could be relegated to supplementary material or a separate review. To take one example, Lines 177-221 describe in detail the resistance and host status of the strains, but this could probably be condensed in order to focus the paper on the more interesting findings presented. In the same content, the number of references 113 seems more appropriate for a review rather than a primary research article (e.g. in Line 83 are all references 12-21 necessary?). The manuscript would be greatly improved by streamlining the results/discussion to emphasize the (truly amazing) points that are discovered in this paper. There are suggestions that the reviewer has made in the “Minor comments” section, but full discretion is given to the authors.

We acknowledge that a number of sections, like Results sections “The inbred strains have diverse and contrasting resistance profiles” (lines 177-221) and “Fitness on different host plants,” were overly detailed to the point of being distracting. We have dramatically reduced both of those lengthy sections in a way that we think still conveys the major points (and they are very important points for the paper, see response to Reviewer 3), and have reduced other sections as well where we provided superfluous details that clouded the main points of the manuscript (for instance, we removed a number of sentences from the Results that should have been in the Methods section only). At the more microlevel, we have systematically gone through every section, including legends, and removed or restructured sentences, and even removed unneeded words within sentences, as possible to reduce length while not impacting meaning.

Against this reduction, we have had to expand some sections moderately to accommodate new analyses and requested additions by all three reviewers (i.e., Results sections “CYPs linked to metabolism of known acaricides are among the genes with strongest trans regulation”).

We have also, as possible, reduced the number of references in the manuscript (but we also had to add several as well in addressing requests by reviewers). The 113 citations in the original manuscript is now reduced to 95 in the resubmission. Including all words in the manuscript (main sections, references, legends, etc.), the resubmission is shorter by nearly 1300 words than the original submission.

*Categorization of gene regulation*

Based on the Figure 3, and the methodology description in lines 816-840, there did no appear to be any FC cutoff for classifying genes as “cis”, “trans” etc. and this distinction is rather based purely on statistical differences in counts between the 5 biological replicates of alleles or parental lines. Are such small changes (<1Log2FC) biologically relevant? Has this approach (No FC cutoff) been used previously?

For initial descriptions of *cis*, *trans*, and other modes of control (like synergism or antagonism), the workflow we used for classification followed other studies, and represents established methods in the field, where no fold change cutoffs are typically applied (see references we cite; for instance, see Figure 3D in the influential McManus et al. 2010 paper, PMID 20354124). Importantly, genes with various modes of control need to be reported across the entire range of fold changes, as this itself provides information about the effect sizes (fold changes) associated with different types of control. This is of basic evolutionary interest.

However, and this is a very ***important*** ***however*** for our study, we are interested in specifically relating differential expression between the inbred strains themselves as we have phenotypic data for the exact strains used in the pairwise comparisons (i.e., to ask how acaricide resistance differences correlate with patterns of differential expression and genetic modes of control). In this context, the specific question of whether “Are such small changes (<1Log_2_FC) biologically relevant?” is admittedly relevant, although it cannot be answered on a gene-by-gene basis (in many or even most cases the answer is very likely no, but in some cases for some types of genes the answer will likely be yes, at some fraction, at least in long-term evolutionary contexts). But, please note that in Fig 4 we did apply parental fold change cutoffs to the results and, for example, we found detoxification genes to be enriched at larger fold changes (this is also illustrated in the right panels in Fig 3 (E-H), where many of the extreme points are D+H+P genes, and we did apply a Log_2_FC > 1 cutoff for those subplots). Notice that the enrichment of D+H+P genes at larger fold changes is only a striking finding because we can perform the comparison across the entire range of fold changes.

Reviewer 2 may also wish to review our responses to a slightly different query about fold changes from Reviewer 3.

Further, the fact that placing such a FC threshold shifts in the proportion of “trans” regulated genes (Lines 314-317; Fig 4) including those present in the detoxification genes (Figure 3 E-H), it appears that mechanisms like “Cis” and “Synergism” play a substantial role in the regulation of gene with large changes. Again the question is of biological relevance, and the reviewer questions whether such an emphasis on “trans” regulation (e.g. the Title) is justified. While several interesting cases are cited of purely “trans” regulation at higher magnitudes (e.g. *Cyp392* family, DOGs), the focus on this within the manuscript seems to minimize the critical contribution of “cis” or “synergistic” mechanisms. It is quite possible the reviewer has missed a key argument, but the authors should address this in the text or explain in the rebuttal why this focus is justified.

In part, there may be a misunderstanding. Briefly, consider the following CYP gene, *CYP392A12*, in the MR-VPi vs. SOL-BEi comparison (lower right corner of Figure 5). Here, there are significant parental, *cis*, and *trans* effects – hence, the genetic mode is classified as **synergism**. However, the log_2_FC approximations for the parental, *cis*, and *trans* effects are +5.5, +0.3, and +5.2, respectively. Notice that the contribution of the *cis* effect to the overall expression effect is tiny (log_2_FC < 0.5), while *trans*-associated upregulation explains nearly all of the expression difference (which is very large for *CYP392A12,* but also for other CYP392A genes).

So, just because there is synergism does not mean that *trans* effects do not dominate in terms of the overall expression differences (as they do for a number of detoxification genes). And, we did mention this in the Discussion (see the last paragraph of section “Multiple modes of genetic control for CYPs known to metabolize acaricides”).

**Having said this**, we did not mean to de-emphasize that *cis* is important, and some D+H+P genes, or other genes for that matter, do show large fold changes for which *cis* contributions dominate. Reflecting this, we have changed the title to read:

“*Trans-driven variation in expression is common among detoxification genes in the extreme generalist herbivore Tetranychus urticae*”

This removes “Abundant” from the original title, which we acknowledge might have bordered on hyperbole, which of course should be avoided. However, the title does still emphasize the observation that we found that *trans* was very common in our study, and for many D+H+P genes with large changes owing to synergism, the *trans* component was often most important (many CYP392 and DOG genes, for example).

Following up on this comment, we added some GO analyses to address a request of Reviewer 3. The GO term enrichments did not change the overall conclusions about the importance of *trans*, but for larger fold changes we did see a few GO terms enriched in *cis* (genes contributing to these enrichments were largely UGTs, which are in the D+H+P gene set). This finding does emphasize as well that *cis* is important (Reviewer 2’s point). Please see the response to Reviewer 3.

On a related issue, it is not clear in in lines 816-840 how categories such as “trans”, “antagonistic”, and “compensatory” were distinguished from one another (only how “cis” and “trans” were distinguished). The authors should edit this to ensure clarify. From Figure 3, which only looks at FC it seems like there are many cases where categories appear to overlap although I assume this is because statistical significance only was used to make such distinctions

We appreciate why Reviewer 2 has raised this comment.

First, we have modified the respective sentence (816-840 in the original submission) to read:

*“From analyses including the detection of differential gene expression between parental strains and ASE in F1s, we assigned seven modes of genetic control (adjusted p < 0.1; see Methods for details). (1) cis only (hereafter cis), (2) trans only (hereafter trans), (3) synergism, where cis and trans act in the same direction on a gene’s fold change with respect to a given parent in a pairwise comparison, (4) antagonism, where cis and trans have opposing effects, (5) compensatory, where there is significant ASE but no differential expression between parents, a result of offsetting effects of cis- and trans-control on expression (a special case of antagonism), (6) conserved (no evidence of expression variation), and (7) ambiguous (where there are discrepancies in criteria used for assignment to the other modes, and for which biological interpretation is unclear).”*

Second, we acknowledge that in the original submission we provided too little detail about how we assigned genetic modes in the Methods. Therefore, to complement the change in the Results (which says “see Methods for details”), we rewrote the respective Methods section “Assignment of mode of genetic control for gene expression variation” to very explicitly describe how the modes were assigned. We do realize that genetic mode assignment is not straightforward to comprehend (it is a very specific, if established, area of study). However, the revised Methods section makes the steps *very clear*, and we want to emphasize that the exact criteria we used to establish the seven modes are very standard in the field (we also cite critical papers in the field in the revised Methods section for readers to turn to if they want more detail).

With respect to Reviewer 2’s second point, yes, the overlap of categories is the result of statistical significance used to make the distinctions. We want to be very clear that this is seen in all such parental/F1 design studies (many of which we cite), for exactly this reason.

**Minor comments: (Reviewer text highlighted)**

*Lines 47-50:* “In particular, a clade of CYPs with documented broad substrate-specificity including multiple pesticides, as well as DOGs that have recently been shown to have broad substrate specificity against plant specialized compounds, were exceptionally highly upregulated as a result of trans effects (or in some cases synergism of cis and trans) in the most multi-pesticide resistant strains.”

*This sentence should be simplified to improve readability*

We have modified the sentence to read:

“*In particular, multiple CYPs and DOGs with broad substrate-specificities for pesticides or plant specialized compounds were exceptionally highly upregulated as a result of trans-regulatory variation, or in some cases synergism of cis and trans, in the most multi-pesticide resistant strains.”*

We also introduced the word “multiple” (i.e., not all) to make it clear that not everything is *trans* regulation, which of course it is not, even if *trans* is major for many key detoxification gene relevant to the acaricide phenotypes we measured (we hope this also alleviates some of Reviewer 2’s concerns that we emphasize *trans* too much).

Lines 137-138: “and a high-quality genome, and is easy to inbreed”.

*Please improve the wording to avoid “and” twice*

This now reads:

*“As opposed to many herbivores, T. urticae has a short life cycle and a high-quality genome [33]. Further, construction of inbred (isogenic) strains by sequential rounds of mother-son crosses is straightforward in this haplodiploid species (males are haploid and females are diploid) [38].”*

The “and” issue is no longer relevant, and we now introduce how inbred (isogenic) lines are made, and that the species is haplodiploid, something that before we did not mention to the end of the Discussion but that arguably should be covered much earlier. This change was also made to address a comment by Reviewer 3.

Lines 162: “revealed 1.19 million high-quality variants”.

*It might be useful to include the density of SNPs in the genome. i.e. 90MB / 1.19 million variants = ~ 1 variant per 800,000 bp?*

To be more specific: 90,000,000 / 1,190,000 = 75.63. So, we have added:

*“… (a variant every ~75 bp….”*

to the sentence. However, this sentence was more generally modified in response to another comment of Reviewer 2 (please see immediately below).

Line 160-170: This can probably be moved to the methods

We agree with the reviewer, and in fact we reduced for first part of the first paragraph of the Results substantially to read:

*“Starting with five outbred strains previously known to vary in acaricide resistance [25,39,40], we performed inbreeding with mother-son crosses for at least seven generations (see Methods). Genome sequencing and variant prediction for the subsequent inbred strains, denoted MR-VPi, MAR-ABi, JP-RRi, ROS-ITi, and SOL-BEi, revealed 1.19 million high-quality SNPs and small indels among strains (a variant every ~75 bp in the 90 Mb genome [33]), a level of polymorphism consistent with prior studies with T. urticae [41–44].”*

And more generally, we moved/removed text from the Results that should have been in the Methods or that was duplicated.

Lines 184-186: “MR-VPi and MAR-ABi, for which progenitor populations were previously reported to be multi-acaricide resistant, exhibited the highest levels of resistance to compounds in multiple acaricide classes (Fig 2A).”

*This sentence is repetitive and should be shortened*

We have simplified the sentence to read:

*“The inbred strains MR-VPi and MAR-ABi retained the high-level, multi-acaricide resistance phenotypes of the progenitor outbred strains from which they were derived (Fig 2A) [25,39].”*

Lines 177-194:

*This paragraph describing the resistance status of each line could probably be shortened or combined. Likewise the following paragraph (194-221).*

We have substantially shortened this entire section (“The inbred strains have diverse and contrasting resistance profiles”) as well as the following section (“Fitness on different host plants”). This was also done to accommodate Reviewer 3’s suggestions.

Lines 244-250 not super necessary

We agree. The respective paragraph in the original submission is now removed entirely in the resubmission.

Lines 256-258: “and because test crosses revealed that viable F1 progeny were readily produced for each of the four crosses (genetic incompatibilities or partial incompatibilities are not uncommon among T. urticae strains [54,70–72]).”

*Were these genetic incompatibilities why the London strain was not chosen or was there another reason that a more standard line could not be used (e.g. mapping bias as it is the reference genome)?*

The primary reason was that the outbred lines used to construct the inbred lines we used were selected to reflect marked variation in resistance to compounds now widely used in the control of *T. urticae* populations. The London strain has also been passed around among labs for many years now, and of course there are risks of contamination. Our sequencing of the five inbred strains we used validated that they were inbred, and the acaricide resistance screening that we did validated that, to a first approximation, the resistance profiles of the inbred lines reflected what was known (or expected) based on the parental outbred lines (it could have been different, but it wasn’t, and had to be established). So, incompatibilities or other reasons were not the specific factor in this case (we were fortunate that incompatibilities did not impact the crosses of SOL-BEi to the other four strains).

Line 278: “For consideration for inclusion in a given comparison”

*Delete “For inclusion”*

In an effort to condense the Results (a concern raised by multiple reviewers), we rewrote the first part of the respective section (section “Prominent trans control of expression variation among inbred strains”). It now reads:

*“Using a parental/F1 experimental design for each of four pairwise comparisons – ROS-ITi, JP-RRi, MAR-ABi, and MR-VPi compared to SOL-BEi, and the respective F1s – we assessed genetic modes of control for genes with polymorphisms (SNPs) in exons, a requirement for detecting ASE in F1s as needed for genetic mode classification. Among genes that were intact and not copy variable (“intact+single copy” gene sets; see Methods for selection criteria), the number….”*

Line 310:

*The reviewer was unable to open Figure S2*

In the PDF version of the submission, we are able to click on the link and download and open the file correctly (we checked all this at the time of submission). We are not sure what to say. Also, the file format is the same as for other files, so we are confused. Perhaps an operating system issue? This would be question for the editorial staff at PLOS Genetics.

Figure 3:

*Perhaps I missed it, but it would be good to clearly state which direction + and – LogFC refers to throughout the ms. E.g. positive numbers always mean enrichment in the unique strains.*

This is a very reasonable request that we agree would make the manuscript more readable. We have placed the following sentence at the end of the second paragraph in section “Prominent *trans* control of expression variation among inbred strains”:

“*Hereafter, with respect to the differential gene expression analyses used in making assignments, positive log_2_ fold change (log_2_FC) values indicate upregulation in parent one (P1, either ROS-ITi, JP-RRi, MAR-ABi, or MR-VPi) as compared to parent two (P2, SOL-BEi), with negative log_2_FC values reflecting downregulation in P1 compared to P2.”*

So, early in the Results, we now make it explicitly clear in a dedicated sentence what the + and – directions refer to, and that they are used throughout.

Finally with this sentence we now also introduce the “P1” and “P2” nomenclature earlier in the manuscript (in the original submission, this was only introduced in the section on inheritance, and the nomenclature is used in several places including in the main text, a figure, and in supplemental tables). This should also help readability.

Line 375 (Figure 5):

*It is unclear how the specific P450s were chosen to for analysis in the phylogeny as only 8 were present in the GO term.*

The second to last sentence in the legend to Fig 5 in the original submission and in this resubmission states:

*“For inclusion of a CYP in the analysis, a genetic mode of control had to be assigned in at least one comparison.”*

But, this is admittedly important enough that perhaps we should have stated it also in the main text. The ending of the first sentence that references Fig 5 now reads:

*“….expression and cis/trans regulation within this gene family (Fig 5; CYPs for which a genetic mode of control was assigned in at least one comparison are shown).”*

Line 400/Discussion: “effects, as observed for the DOG genes DOG1, DOG11, and DOG13 (in MAR-ABi) (S3 Fig), the lipocalins”

*Are these genes which show similar trans regulation close to each other physically on the chromosome. Could they possess common regulatory factors?*

We hope we understand the question. But if, however, the reviewer is asking if adjacent genes are controlled by the same *cis* element (which might allow the binding of a *trans*-factor to simultaneously regulate adjacent genes), that is theoretically possible in some cases (either in the context of a possible locus-control region, but there are relatively few documented cases of this, or of duplicated genes where *cis* elements are duplicated along with coding sequences). However, although many genes in eukaryotic genomes do arise next to each other via tandem duplication (and are physically close), including in *T. urticae*, many genes we highlight in our study are not (for instance, *CYP392A11* and *CYP392A16* are not physically close in the genome, but each show major and similar *trans* effects, see Fig 5).

Line 494-496: “with dominant versus additive inheritance closely mirrored the higher frequency of trans versus cis effects (this was true among comparisons as well; for instance, for the ROS-ITi x SOL-BEi contrast, relatively fewer trans effects and dominance were detected as compared to the other three, compare Fig 3A-D and Fig 4A to Fig 6A).”

*No need for parentheses, just make this an additional sentence.*

We agree, done.

Line 555-574:

*Cap’n’collar is probably worth a mention in the context of potential trans acting factors as it has been widely cited as a transcriptional regulator in insecticide resistance.*

We have now restructured the third paragraph of Discussion section “Enrichment for *trans* regulation among detoxification genes” to mention Cap‘n’collar C, and we cite a review of potential regulators of detoxification gene expression that may be helpful for readers wishing to learn more about this area. In particular, please see the following sentence we added to this section:

*“In insects, several transcriptional regulators have been implicated in responses to xenobiotics, including genes in the Cap’n’collar isoform C and HR96 families (see Amezian et al. [65] for a recent review). Although little is known about regulators of detoxification pathways in T. urticae, the genome encodes multiple HR96 family members [41].”*

Lines 576-611:

*The authors could probably shorten discussion of specific P450s*

We have shortened this section (section “Multiple modes of genetic control for CYPs known to metabolize acaricides”).

Lines 724-725: “we used the approach of Villacis-Perez et al. [54].”

*The authors should briefly explain the approach of Villacis-Perez.*

What we did here was actually fairly straightforward. But, the methods sentence has been modified to read:

“*Further, we applied the approach of Villacis- Perez et al. [54] to assess if the inbred strains were in fact genetically isogenic; briefly, by strain and SNP site, we used alternative and reference allele count data in VCF files to assess the fraction of SNPs supported by only one allele (i.e., the fraction that were fixed, an expectation for isogenic strains).”*

Further, and this may not have been clear, but the findings of this analysis were presented in the original Results section, first paragraph, in the sentence that reads:

*“Further, more than 98% of SNP sites were fixed in each of the five strains, confirming earlier reports that ~7 generations of inbreeding in this haplodiploid species results in isogenic (or nearly isogenic) strains [41].”*

This sentence is retained in this resubmission.

Reviewer #3: The paper by Kurlovs et al. targets an important question in insect genomics, “how transcriptional changes are regulated”? The presented work looks to me quite pioneering in many aspects and therefore, I do think that the paper should be eventually published in PLOS Genetics. However, at least to my opinion, I think that more bioinformatic work should be conducted and the paper should also go through a process of condensation and focusing. Below I outline 8 major comments followed by few additional comments.

We thank the reviewer for the appreciation and constructive comments. As described above in the responses to Reviewer 2, we have condensed and focused the manuscript.

General/major comments:

I. I have read the abstract and introduction several times and still could not figure out what exactly is the scientific question. Is it general transcription regulation in Tetranychus urticae (how the genome of this spider mite works?), is it how transcription is regulated in response to acaricides selection works, to plant hosts selection? Is it only a CYP, DOG paper? What exactly are we studying so one can judge if the right experimental system was established.

The study looks at the genetic control of gene expression variation in five inbred (and isogenic) lines that we show, in sections “The inbred strains have diverse and contrasting resistance profiles” and “Fitness on different host plants,” vary markedly in acaricide resistance and also vary in performance on different hosts. Thus, transcriptional variation between strains potentially underlies the phenotypic variation in these strains against major selective agents; on top of this, we address the genetic basis of the expression variation. The intellectual questions are laid out in the Introduction, including the ending of the 4^th^ paragraph that now reads as follows:

*“The extensive and rapid transcriptional reprogramming associated with the exceptional adaptation potential of T. urticae raises several questions about the mechanisms of gene regulation in polyphagous herbivores. Given the often very large population sizes of this species in agricultural settings, selection acting on cis variants at many loci is plausible. However, the often coordinated and rapid responses in gene expression patterns observed between strains after selection by both acaricides and host plants [24,25] raises the alternative possibility of selection acting on trans-acting factors that serve as master regulators of many genes in detoxification/adaptation pathways that are located throughout the genome.”*

And then, our study addresses the questions that are raised. With respect to the question “Is it only a CYP, DOG paper?” – no, the study uses carefully selected and developed biological materials and experimental designs to assess the types of genes harboring expression variation, and their genetic modes of control, that associate with the documented phenotypes (and that hence may underlie resistance phenotypes in *T. urticae*). We found that CYPs and DOGs were at the extremes of expression variation, but going into the study we did not know what we would find (so that is a major finding that came out of the study itself). Of course, because of the extreme interest in CYPs especially, it would have been odd to not give some discussion to them even if the results had turned out not to be as striking (and we carefully introduce this in the Results section titled “CYPs linked to metabolism of known acaricides are among the genes with strongest *trans* regulation”).

Our license to change the Abstract and Introduction is very limited, as Reviewer 1 noted that “the manuscript is very well written” and, while Review 2 had a number of suggestions, only minor revisions to the Abstract and Introduction were requested (which we addressed, see responses to Reviewer 1 and 2). Nevertheless, we have made small changes to the Introduction to address Reviewer 3’s comments as described below.

II. While “cis” regulation is quite easy to understand, “trans” regulation is not. I think the authors should do much better work in the introduction to explain what “trans” regulation is all about (up to their mode of action). This cannot wait until the discussion because it significantly interferes with the understanding of the results part.

In response also to Reviewer 2, we changed the first part of the second paragraph of the Introduction to read:

*“One widely used approach to understand the genetic control of gene expression variation in diploid organisms, as well as its associated inheritance, involves comparing expression between parents and F1 offspring [11–13]. If a difference in gene expression between two parental inbred strains is due to cis effects only (i.e., changes in promoter or enhancer regions that influence gene expression on the same chromosome), the same difference ratio will be observed for the respective parent-of-origin transcripts in F1 offspring (i.e., allele-specific expression, or ASE; parent-of-origin for transcripts can be assigned using RNA-seq when variants are present in transcribed sequences). However, if the ratios for expression differences between parents are not the same as for F1 ASE ratios, trans effects – which reflect variation in diffusible factors such as transcription factors or components of upstream signaling pathways that can impact the expression of multiple target genes on both chromosomes – contribute to expression variation [8].”*

We now further explain both *cis* and *trans*, and we formally introduce that *trans* acts via a diffusible factor and *cis* does not (part of the well-recognized formal definitions) and that variation acting on *trans* factors can impact multiple genes, including the same copies of genes on both chromosomes.

Additionally, we changed the last sentence of the 4^th^ paragraph of the Introduction from:

*“However, the often coordinated and rapid responses in gene expression patterns observed between strains after selection by both acaricides and host plants [33,34] raises the alternative possibility of selection acting on trans-acting factors that serve as master regulators of detoxification/adaptation pathways.”*

to now read:

*“However, the often coordinated and rapid responses in gene expression patterns observed between strains after selection by both acaricides and host plants [33,34] raises the alternative possibility of selection acting on trans-acting factors that serve as master regulators of many genes in detoxification/adaptation pathways that are located throughout the genome.”*

This change emphasizes that variation in a *trans* factor can impact expression of many genes across the genome (which should help in enforcing an understanding of *trans* effects).

We also note that *cis* and *trans* are very well understood and developed concepts for the anticipated readership of PLOS Genetics, although we agree that a lengthier explanation would be beneficial for a non-genetics focused, specialized journal.

III. Methods section lines 652-667 (mite strains) raises few questions. We learn that three strains (maybe even four) have a history of selection to acaricides but we don’t know if this selection continues or not. We also do not know how long the strains were maintained on bean plants (since their collection or only before the experiment?). These two are concerns because we do not know exactly what the authors are looking for and if comparing genomes that were heavily selected versus ones that were not is the right way to go for asking questions on how genomes work?

A few points are raised here, and we also think that there has been a misunderstanding that may have resulted from our unclear wording of this section. We have now clarified that the acaricide selection did not occur during inbreeding, or after inbreeding, and that the strains were maintained on bean throughout the work covered in the manuscript (excepting, of course, the host plant shifts, see Results section “Fitness on different host plants”).

Resistant progenitor strains like MAR-AB and MR-VP have a history as laboratory strains with strong pesticide selection; their selection histories and host histories are carefully described in the studies we cite (but, they were maintained on bean in lab). However, these were non-inbred strains, meaning that they were segregating for genetic variation. Once we made the inbred lines, the critical finding is that the resulting inbred lines showed acaricide resistance variation (and also variation on different hosts). **This is the key finding of relevance**, because only the derived inbred strains are used in the current study, and once an inbred (isogenic) strain is produced, it cannot change genetically (or at least only by new mutation, which all studies like ours presume is negligible, and it is a very reasonable assumption).

Finally, please note that we don’t know the acaricide selection history of the strains BEFORE they were collected, or the long-term history of their hosts before they were collected (and most *T. urticae* populations almost certainly move among hosts in time frames of months to years). Again, what is most important is that we showed that the inbred strains we are using now, in this study, do vary in acaricide resistance and host plant use (Fig 2). The fact that the inbred strains derived from the outbred multi-acaricide resistant strains MAR-AB and MR-VP maintained resistance to so many compounds was reassuring, of course.

To clarify the above concerns, we have modified the first section of the Methods to read:

*“Inbred strains used for genetic and gene expression analyses originated from five T. urticae strains that we maintained on potted bean plants or detached bean leaves on wet cotton (Phaseolus vulgaris var. ‘Prelude’): ROS-IT, MAR-AB, MR-VP, JP-RR, and SOL-BE. Briefly, SOL-BE was collected on potato in Belgium in 2018, and ROS-IT was collected from roses in Italy in 2017. The MAR-AB strain originated from a heavily sprayed greenhouse near Athens (Greece) in 2009, and is resistant to abamectin, bifenthrin, clofentezine, hexythiazox, fenbutatin oxide and pyridaben [25]. The MR-VP strain originates from a lab stock originally collected in September 2005 from bean plants in a greenhouse in Brussels, Belgium, and was selected for its extraordinary levels of resistance to mitochondrial electron transport inhibitors of complex I (METI-Is) [39]. The laboratory strain JP-RR was selected for its resistance to electron transport complex II (METI-II) inhibitors (cyenopyrafen, cyflumetofen, and pyflubumide) from its parental strain JP-R, as described by Khalighi et al. [40]. To avoid the confounding effect of heterozygosity on downstream studies of the control of gene regulation, the strains were inbred via mother-son mating performed on detached bean leaves as described by Bryon et al. [38] for 7 to 9 generations to produce the respective inbred strains (post-fixed with the “i” for inbred designation; ROS-ITi, MAR-ABi, MR-VPi, JP-RRi, and SOL-BEi). After the final round of inbreeding, the strains were expanded and maintained on potted bean plants to produce sufficient mites for downstream analyses (no acaricide selection was applied either during or after inbreeding). All strains were maintained at 25°C (±0.5°C), 60% relative humidity, and 16:8 h light:dark photoperiod.”*

IV. According to lines 664-665 it can be understood that each inbred strain is from one female. How many inbred strains per parent strain? How did you control for genetic drift? How many inbred strains were used per parent strain in performance and expression assays? This unclarity comment goes throughout the manuscript. Meaning, when we read that DNA was isolated from 400 adult females (line 691), are they all progeny of one female? Similarly, for the crossing experiments, were all 40 males and 40 females of each inbred strain from the same mother. If yes, how this can match the variance in the parental strain, if no, how was the work conducted?

A major source of confusion was, unfortunately, introduced by our poor wording (which has now been corrected, see below). At the end of the series of mother-son inbreeding steps that each go through a single mother (female), expansion on plants was performed to generate enough mites for DNA collection, and for the crosses used to produce RNA, etc.

But, at this step, the strains were inbred. That is the key point. So, all mites in a given strain were genetically identical (and we validated this by whole-genome sequencing). In an isogenic strain, there is no such thing as genetic drift, etc. Whether DNA was collected from females from one mother mite of an inbred strain, or from those of multiple mothers, does not matter as the strains were isogenic – the DNA is all the same (to explicitly answer the question for the purpose of this reviewer response, the 400 females were from more than one female as a single *T. urticae* female cannot lay 400 eggs). We are sorry about this confusion. The second to last paragraph of Methods section “Mite strains” now reads:

*“After the final round of inbreeding, the strains were expanded and maintained on potted bean plants to produce sufficient mites for downstream analyses (no acaricide selection was applied either during or after inbreeding).”*

Additionally, the first sentence of the last paragraph of the Introduction is now split into two, and reads:

*“As opposed to many herbivores, T. urticae has a short life cycle and a high-quality genome [33]. Further, construction of inbred (isogenic) strains by sequential rounds of mother-son crosses is straightforward in this haplodiploid species (males are haploid and females are diploid) [38].”*

This now clarifies, early in the manuscript, that inbred (and hence ***isogenic***) strains can be made easily in *T. urticae*. As an aside, construction of isogenic strains is very hard to do in most animals; it works well in *T. urticae*, presumably because highly deleterious alleles (like lethal mutations) that impede inbreeding are not likely to accumulate in populations as they are removed in the haploid sex where their highly deleterious action is unmasked). The change also makes it clear why use used females in the study for RNA-seq. Females are the diploid sex, a requirement to detect ASE, and females are much larger than males and therefore are the sex that causes by far the most damage to plants in agricultural and natural settings (mite populations tend to also be female sex biased).

V. Two paragraphs in the results (177-221 and 223-240) do not belong (at least to my opinion) to this paper as they do not aim to understand transcription regulation or contribute to the parents-F1 comparisons for determining “cis”, “trans” or “other” regulation. I would like to note that the first aforementioned paragraph is the best I read on genomics of acaricide resistance but it simply belongs to a different story (manuscript).

As described above, it was critical that we present data to show that the inbred (isogenic) strains we produced maintained the acaricide resistance phenotypes of the outbred strains from which we derived them (or showed variation in resistance to compounds that were not previously tested). The same goes for host plant use. Briefly, if strains are not established to vary for acaricide resistance and host plant use, than any resulting transcriptome results are not relevant for the phenotypes in question (resistance and host use in this case given *T. urticae*’s life history). The same would hold true if the apparent resistance resulted only from target-site changes (which is not what we observed, suggesting transcriptome changes are involved!).

However, we acknowledge that the two sections were far too long and wordy, and distracted from flow of the manuscript. Therefore, we shortened them *significantly*. Please see our response to Reviewer 2, who did not ask that the sections be removed, but who did ask that they be shortened substantially (which, again, we have now done, please see the revised manuscript).

VI. At the end, parents-F1 comparisons were made only using bean as a host plant (the one on which performance is best according to Figure 2B) with no acaricide treatment. This is fine but it reflects only this specific combination. We do not know if the regulation of many genes will stay similar on more challenging hosts for example (tomato) or under acaricide treatment.

Reviewer 3 has picked up on a very interesting question. The scope of our study, including the number of comparisons and the number of replicates, is more than for many comparable studies, and expanding this design to potentially many different acaricide selection scenarios and many hosts is challenging. But, this is an obvious area of future study. We believe our work is an important first step toward a broader understanding of how the environment (including acaricides and hosts, the chemical environment) impacts the genetic control and inheritance of gene expression. Our study also further establishes *T. urticae* as a system in which these and other questions can be asked in future. Please note as well that the last sentence of the Discussion now ends:

*“More generally, our findings position T. urticae as an attractive system for future studies to characterize the molecular basis of the control of gene expression variation in xenobiotic and host plant use pathways.”*

VII. There is something I do not understand in the logic or basics of the work. According to Table S4, the authors had discovered a gold mine of nearly ~7,680 genes, from which 51.9% to 66.4% fell into interesting regulation categories. Instead of conducting enrichment analyses for identifying major pathways, families etc, they focus most of their efforts on a relatively short list of about 470 genes mainly from the detox families. I don’t think that studies trying to understand how genomes function should limit themselves to such a narrow observation which covers only about 6% of the analyzed genes. In some way it feels like looking for the answer only where there is light or staying in the comfort zone. To my opinion, the authors should at least provide a list of the major enriched functions/families using all the data and their generalized mode of regulation.

We appreciate why the reviewer requests these additional analyses. We do focus initially on detoxification / host / peptidase associated genes (Fig 3E-H and Fig 4B). This is driven by our interest in these families, which we would argue is reasonable given our selection of study system and questions.

However, in the original submission we also performed GO enrichment analyses on the top 2.5% tails of extremes of expression variation in *cis* and *trans*. This analysis was independent of a priori assumptions about detoxification genes (see the following sentence in our original submission: “*In a complementary approach independent of a priori gene family classification, we also investigated the molecular nature of genes with high differential expression as a function of their genetic control mode.”).*

At Review 3’s request, we have now expanded the GO enrichment analyses in two ways.

First, for each of the four comparisons, we performed GO enrichment analyses for all genetic modes (excepting category “ambiguous”). Using all genes, only a few GO terms were enriched, and there was little pattern across comparisons. As part of this analysis, we then also imposed the log_2_FC cutoffs presented in Fig 4, and for genes by genetic mode where log_2_FC > 2 between parents there was a consistent pattern of enrichment. Perhaps not surprisingly, based on the prior sections and our original analyses (first submission), these were for the *trans* and synergism categories for terms associated with CYPs and DOGs (but, this analysis is what the review requests, and we do believe it is so obvious to do that we should have included it in the original submission, so that was an oversight on our part).

Second, we repeated the enrichment analyses for the *cis* and *trans* distributions, but we were modestly less restrictive in doing so. We took genes in the 5% tails of the distributions as assembled across all four comparisons. For this analysis, and the one above, we also used a different (and more appropriate) GO enrichment method, as described below. With this analysis, we recovered a modestly larger number of GO terms as compared to the original submission. Reassuringly, our finding that terms associated with CYPs and DOGSs were enriched at both extremes of the *trans* distribution replicated. In addition, a modest number of other GO terms were now enriched in *trans* – genes that contributed to this enrichment were SDRs (mentioned in the original submission), but now also UGTs featured prominently (another detoxification gene family, but that is the result, and it is interesting). In the original submission, we found no enrichment of GO terms in the extremes of the *cis* distribution. This is now changed, however, as two GO terms were enriched in *cis* extremes of fold change, driven in large part also by UGTs and SDRs. However, most enriched GO terms were nonetheless for *trans*, as before. Finally, several GO terms represented by genes not in the D+H+P gene set were now also identified for extremes of *trans*, including terms associated with the cuticle biology. We report these, as it is a finding (and possibly an important one), but we do hope the reviewers appreciate that not every GO term can be discussed (and as the reviewers noted, the manuscript is long).

The new analyses associated with Review 3’s request have been incorporated at the beginning of section “CYPs linked to metabolism of known acaricides are among the genes with strongest *trans* regulation”, the same place that the original GO term analyses were presented (much of the first half of this section is rewritten, please see the resubmitted manuscript). We also made some changes to the second half of this section to incorporate the changes, and to shorten this long section where possible (a general request of Reviewer 2 and 3).

Associated with these analyses, of necessity we rewrote Methods section “Gene ontology (GO) enrichment analysis.” When we redid these analyses, we used a more standard way to assess GO term enrichments than we did in the original submission (we now use the enricher function of the clusterProfiler v4.2 package in R, which is widely accepted for this type of analysis).

We thank Reviewer 3 for prompting us to do these analyses, and believe the revisions add to the manuscript. Associated with the changes to the main text, a new supplemental table was added (S7 in the resubmission), and what was S6 Table in the original submission is now S8 Table with the expanded analysis.

VIII. What also is missing to my opinion is something beyond “cis”, “trans”, dominant, which is nice but does not really give a mechanistic explanation to regulation beyond the general feeling the genomic background matters (we kind of know that already). For example, the first time the term “transcription factor” appears is in line 564 (deep in the discussion). Why not put some efforts in running co-expression network, for the identification of modules and hubs and relate some of the discussion to the observed findings and potential discovery of regulatory elements?

We can understand Reviewer 3’s sentiment, but would raise several points. We assumed that, for a leading genetics journal, transcription factors would be understood as potential players in the mechanics of regulation, but we agree to now state so in the Introduction (where we now also briefly define the well-known terms of *cis* and *trans* regulation in more detail). We do believe, however, that for geneticists understanding “genetic background” as termed above does matter, a lot. We would also argue that our finding of a substantial role for *trans* in detoxification gene expression regulation substantially extends knowledge for the field; it especially contrasts to other studies that have often focused on isolated if dramatic instance of *cis* regulation at specific detoxification genes (see the Discussion). Additionally, we would hope that Reviewer 3 can appreciate that the requested additional studies would dramatically increase the length of the manuscript, if they were even successful (which is a question given the small sample sizes combined with the genetic structure of crosses to a common parent, which would introduce caveats). This is at odds with the requests of two of the three reviewers, including Reviewer 3, to condense the manuscript (which, again, we admit is on the longer side).

Other comments

General:
The paper is a bit too long. Beside considering focusing only on transcription regulation (removing the acaricide resistance and host performance chapters), please condense by “cleaning” “methods” and “discussion” parts that appear extensively in the “results” section.

We agree. We have substantially shortened a number of paragraphs and sections, especially section “The inbred strains have diverse and contrasting resistance profiles” and the following section “Fitness on different host plants”. For additional details about how we have reduced the manuscript, please see our responses to Reviewer 2.

Abstract
Lines 43-44 – Most readers read the abstract before reading the whole manuscript. With this respect, it is not clear what the authors mean by “trans” instead of “cis” which is explained only in the introduction.

The three reviewers might have noticed that we specifically defined “acaricides” as we thought the general readership for PLOS Genetics might not know this term. Likewise, if this manuscript was being sent for review at a specialized journal unrelated to genetics, we agree with the reviewer that defining *cis* and *trans* in the abstract might be prudent.

But, *cis* and *trans* are well understood in the genetics community, and in genetics-focused journals are not usually defined explicitly in the abstract (at least not within the last decade or so). The following are some of many examples: Nature Genetics (Shi et al., 2012; PMID 22805557), Genome Research (Coolon et al., 2014; PMID 24567308; Goncalves et al., 2012; PMID 22919075), The Plant Cell (Springer & Stupar, 2007; PMID 17693532), eLife (Albert et al., 2018 PMID: 30014850), and The Plant Journal (Ji et al., 2022; 35575017).

Also, the word limit for abstracts in PLOS Genetics is strict. However, we do define *cis* and *trans* near the very beginning of the Introduction (the start of the second paragraph), and as described above we have elaborated on the definitions in this resubmission (see also response to Reviewer 2).

Lines 84-86 – Shouldn’t you be sharper here? Maybe I am wrong here but it is not just changes in promoter or enhancer regions, it is the promoter or enhancer regions physically linked to the expressed allele.

Yes, we could have been sharper in defining *cis* and *trans* and how such regulation works and can be detected (see also response to Reviewer 2). The respective sentences in the Introduction now read (beginning of the second paragraph):

*“One widely used approach to understand the genetic control of gene expression variation in diploid organisms, as well as its associated inheritance, involves comparing expression between parents and F1 offspring [11–13]. If a difference in gene expression between two parental inbred strains is due to cis effects only (i.e., changes in promoter or enhancer regions that influence gene expression on the same chromosome), the same difference ratio will be observed for the respective parent-of-origin transcripts in F1 offspring (i.e., allele-specific expression, or ASE; parent-of-origin for transcripts can be assigned using RNA-seq when variants are present in transcribed sequences). However, if the ratios for expression differences between parents are not the same as for F1 ASE ratios, trans effects – which reflect variation in diffusible factors such as transcription factors or components of upstream signaling pathways that can impact the expression of multiple target genes on both chromosomes – contribute to expression variation [8].”*

Line 93 – “versus” is confusing. Do you mean “over”?

We have modified the sentence to read:

*“For example, in many instances, expression variation more often results from variation in trans regulation compared to cis regulation in intraspecific comparisons…”*

Line 96 – Isn’t it more linked to the mode of action of the trait?

We have modified the sentence to now read:

*“Further, for genes for which cis effects explain expression variation, additivity in inheritance is often observed; in contrast, genes for which variation is explained in trans are usually enriched for dominance [12–16].”*

We hope that we understand Reviewer 3’s point correctly, and that the revised wording addresses the concern.

Lines 108-109, 127-130 – I would be more carful here. Adaptation to insecticides and to host plants work at very different time scales and host plant selection and utilization is far more complex than surviving a lethal dose of an insecticide. I am not sure that short-term responses to a host plant (which most studies test), actually reflect meaningful evolutionary processes of host adaptation.

We understand Reviewer 3’s take on this point, and this would certainly be true for specialist herbivores. However, it is likely relevant for polyphagous organisms that are known to adapt to different hosts in only a moderate number of generations (we provide citations for studies suggesting that adaptation can occur in *T. urticae* in as few as five generations, which might be the length of time a *T. urticae* population may find itself on a single host in a growing season).

Line 139 – Delete “and”.

Thanks for pointing this out. This sentence has now been split (the inappropriate use of “and” is resolved):

*“As opposed to many herbivores, T. urticae has a short life cycle and a high-quality genome [33]. Further, construction of inbred (isogenic) strains by sequential rounds of mother-son crosses is straightforward in this haplodiploid species (males are haploid and females are diploid) [38].”*

Results:
Line 172 – Aren’t there five inbred strains it total?

Yes, there are five. We see the source of confusion in how the sentence can be read, and have modified/simplified it to just read:

*“Our PCA using all strains confirmed De Beer et al.’s [45] observation that SOL-BEi and MAR-ABi are genetically distinct, and SOL-BEi was distinct from the other inbred strains as well.”*

Lines 292-301 and also “Methods” lines 819-823. I think I understand how “cis” and “trans” were assigned. I don’t think it is explained how expression patterns to the other categories were assigned. Also, you have six categories in the “results” and seven in the “methods”.

Reviewer 2 also had questions about the assignments, and we should have explained the designations and their derivations much more carefully (our apologies). Please see our detailed response to Reviewer 2. But, briefly, the second paragraph of Results section “Prominent *trans* control of expression variation among inbred strains” now reads:

*“From analyses including the detection of differential gene expression between parental strains and ASE in F1s, we assigned seven modes of genetic control (adjusted p < 0.1; see Methods for details). (1) cis only (hereafter cis), (2) trans only (hereafter trans), (3) synergism, where cis and trans act in the same direction on a gene’s fold change with respect to a given parent in a pairwise comparison, (4) antagonism, where cis and trans have opposing effects, (5) compensatory, where there is significant ASE but no differential expression between parents, a result of offsetting effects of cis- and trans-control on expression (a special case of antagonism), (6) conserved (no evidence of expression variation), and (7) ambiguous (where there are discrepancies in criteria used for assignment to the other modes, and for which biological interpretation is unclear).”*

This resolves the issue of six versus seven categories (there are now seven explicitly defined in both the Methods and Results).

Further, we have restructured/rewritten the Methods section “Assignment of mode of genetic control for gene expression variation” to very clearly define how the modes were assigned. Although there could be quibbles about the exact logical flow in making assignments, **we want to be extremely clear that we use the established workflow that is widely accepted in the field** (and we give appropriate references). Again, for more detail, see our response to Reviewer 2.

Line 294 – As this is quite a relaxed significance level, I would also add a filter on fold-change, especially in relation to the findings in lines 315-317. You do not want “noise” to affect too much your conclusion on the dominance of “trans” regulation (you can go to 1.5-fold, although I think that 2-fold is preferable, but I would not recommend lower than that).

Our study was highly powered to detect differential expression, as for most comparisons we have 5 biological replicates (few studies like ours do that). Therefore, many genes with small to modest differences in fold change in expression are significant (see S4 Table). **However, for what are potentially biological reasons, the fraction of *trans*-regulated genes as a function of fold-change can vary.** For example, *trans*-regulatory variants of large effect that cause large expression changes in many downstream genes may be particularly deleterious, leaving only those with more moderate (but nonetheless real) effects to persist in populations (and *cis* changes of large effect – i.e., resulting in large fold change differences – may not be selected against strongly as only one gene is impacted). Such scenarios have been discussed in the literature. So, in this instance, a fold change cutoff may impact biological interpretations.

What we think is a better way to assess if the significance level we selected impacted the general conclusion of more *trans* than *cis*, is to evaluate with a more stringent p-value cutoff (not fold change cutoff, because, again, that might mask biologically relevant phenomena). We actually did this already just to be reassured about our conclusions; below find the number of genes by comparison regulated in *trans* versus those in *cis* for a 0.001 adjusted-p value cutoff. The results with adjusted-p < 0.1 are also shown for comparison (from Figure 4A-B):

**ROS_IT vs. SOL-BEi comparison:**

Adj p < 0.1:	trans =1241,	cis = 736,	**% trans = 0.63**

Adj p < 0.001:	trans = 576,	cis = 440,	**% trans = 0.57**

**JP-RRi vs. SOL-BEi comparison:**

Adj p < 0.1:	trans = 1970,	cis = 609,	**% trans = 0.76**

Adj p < 0.001:	trans = 1305,	cis = 434,	**% trans = 0.75**

**MAR-ABi vs. SOL-BEi comparison:**

Adj p < 0.1:	trans = 1804,	cis = 645,	**% trans = 0.74**

Adj p < 0.001:	trans = 1026,	cis = 417,	**% trans = 0.71**

**MR-VPi vs. SOL-BEi comparison:**

Adj p < 0.1:	trans = 1810,	cis = 627,	**% trans = 0.74**

Adj p < 0.001:	trans = 1154,	cis = 385,	**% trans = 0.75**

With the *very strict cutoff of Adj p < 0.001*, the ratio of *trans* to *cis* control changes only slightly, even though the numbers of genes by category are reduced (which of course is expected when using such a small *p*-value; in one case, for the MR-VPi vs. SOL-BEi comparison, the ratio of *trans* to *cis* actually went up). So, although we have not presented a full analysis in the manuscript (presenting data this complex over multiple p-value cutoffs would be a challenge in practice), we did the above check to assess the robustness of our findings.

The specific findings above are captured in a new supplementary table, S5 Table, in the resubmission. To introduce this finding into the main text, the respective sentence in the 3rd paragraph of section “Prominent *trans* control of expression variation among inbred strains” now reads:

*“Among all genes for which a genetic mode of control of cis, trans, synergism, antagonism, or compensatory could be assigned, those with trans control were more numerous by between 2.80- to 3.23-fold as compared to the second most frequent category (cis), excepting for the ROS-ITi × SOL-BEi comparison (1.69-fold, Fig 3A-D; the preponderance of trans compared to cis control was observed even when a much more restrictive adjusted-p value, 0.001, was used for assigning cis and trans modes, S5 Table).”*

Discussion:
Lines 541-543 – This example is not clear.

In an effort to condense the manuscript, we have now deleted this sentence, and the one after, from the manuscript.

Methods:
Line 734 – confusing with line 729. Do you refer here to collection for RNA extraction?

Yes, we see the issue, thanks. To clarify what the crosses and resulting mites were used for, the second to last sentence in this section has been changed to:

*“For each replicate (F1 or parental), 100-120 adult female mites at four days of age were collected for RNA extraction, except for the JP-RRi x SOL-BEi F1s for which females of 4-7 days old were collected.”*

(we added “for RNA extraction” to the sentence)

********************************

Additional comments:

1. For the D+H+P gene set, in our original submission we incorrectly stated how we obtained digestive peptidase genes. The respective sentence in Methods section “Detoxification and host plant associated genes” now reads:

*“For putative digestive proteases, we focused on the large C1A (papains, cathepsin L) subfamily of cysteine peptidases as listed in column one of Grbić’ et al.’s [33] supplementary table “Table S6.1.9. Cysteine peptidase genes in T. urticae” (note that the tetur13g02490 identifier was replaced by tetur13g02500 in later genome annotations).”*

Briefly, the tables in the Grbic et al. manuscript we mentioned in the original submission included peptidases that are likely not involved in digestion of plant tissue (like caspases involved in programmed cell death), and that we did not use in the analysis. We apologize for this Methods section oversight that has now been fixed.

2. We made an inadvertent mistake in parsing the information presented in S7 Table in the original submission. This did not impact any analyses, or Fig 6 – again, it was in preparing the S7 Table itself (the intact+single copy genes by strain were not resolved). In the resubmission, this is fixed (note that what was S7 Table is now S9 Table in the resubmission). To S9 Table, we now also added mean expression values as well, which we should have done earlier as these values were also used in the workflow to generate inheritance modes. Apologies for any confusion and the issue is fixed; again, no analyses or results were impacted, it was just the supplementary table itself.
